# Deep learning for crown profile modelling of *Pinus yunnanensis* secondary forests in Southwest China

**DOI:** 10.3389/fpls.2023.1093905

**Published:** 2023-02-03

**Authors:** Yuling Chen, Jianming Wang

**Affiliations:** ^1^School of Environmental and Resources Science, Zhejiang A & F University, Hangzhou, China; ^2^Department of Mathematics and Computer Science, Dali University, Dali, Yunnan, China

**Keywords:** crown profile, LSTM and its variant algorithms, LightGBM, SHAP, *Pinus yunnanensis*

## Abstract

Accurate information concerning crown profile is critical in analyzing biological processes and providing a more accurate estimate of carbon balance, which is conducive to sustainable forest management and planning. The similarities between the types of data addressed with LSTM algorithms and crown profile data make a compelling argument for the integration of deep learning into the crown profile modeling. Thus, the aim was to study the application of deep learning method LSTM and its variant algorithms in the crown profile modeling, using the crown profile database from *Pinus yunnanensis* secondary forests in Yunnan province, in southwest China. Furthermore, the SHAP (SHapley Additive exPlanations) was used to interpret the predictions of ensemble or deep learning models. The results showed that LSTM’s variant algorithms was competitive with traditional Vanila LSTM, but substantially outperformed ensemble learning model LightGBM. Specifically, the proposed Hybrid LSTM-LightGBM and Integrated LSTM-LightGBM have achieved a best forecasting performance on training set and testing set respectively. Furthermore, the feature importance analysis of LightGBM and Vanila LSTM presented that there were more factors that contribute significantly to Vanila LSTM model compared to LightGBM model. This phenomenon can explain why deep learning outperforms ensemble learning when there are more interrelated features.

## 1 Introduction

*Pinus yunnanensis* is a major component of coniferous forests in southwestern China. It has been extensively cultivated for reforestation and ecological engineering (Sun et al., 2008). In southwest China, it occupies approximately 52% of the forested area and produces 32% of the timber volume (Jin and Peng, 2004). As a pioneer tree species, *Pinus yunnanensis* is shade-intolerant, deep-rooted, drought-resistant, and tolerant of rocky soils with low fertility (Xu et al., 2016). It plays a crucial role in regional economic development and ecological restoration (Wu, 1986; Jin and Peng, 2004; Xu et al., 2016). Knowledge of the dynamics of *Pinus yunnanensis* promote the regeneration of secondary forests and further help China to better achieve its carbon neutrality target in 2060 because it can effectively improve forest carbon sinks in the region (Deng et al., 2022).

Crown profile is mostly related to the competition of individual trees in the stands, light interception, growth, and yield of trees. However, crown measurement is time-consuming and labor-intensive. It is impossible to measure the crown of every tree in actual production, so it is necessary to build the high-precision crown profile models. Crown profile models are key components of growth and yield models in the evaluation of competition among trees, forest microclimate, and biodiversity (Dong et al., 2016; Sun et al., 2022). Accurate information concerning crown profile is critical in analyzing biological processes (e.g., photosynthesis, stand growth, survival, and competition) (Rautiainen et al., 2008; Dong et al., 2016; Wang et al., 2021), whilst providing a more accurate estimate of carbon balance (Plesoianu et al., 2020; Yang et al., 2022). In mixed species ecosystems, modeling individual specie crown profile models is necessary if seeking to analyse species-specific phenological trends, plasticity, and responses to extreme events (Fawcett et al., 2021).

In forest surveys, the crown profile database contains multiple measurements for each sample tree crown, which displays hierarchical structural features. Crown profile models were initially fit assuming that the within equation errors were independent and identically distributed. However, since crown profile models in that several measures are taken on each crown profile, autocorrelation among measures within a profile is likely. For linear statistical models, the least squares estimate of regression coefficients remain unbiased and consistent in the presence of autocorrelation, but they are no longer efficient (Myers, 1990; Crecente-Campo et al., 2009). It has been of interest for forest modelers to better understand this phenomenon, particularly on the basis of statistical modeling and analysis (Wang et al., 2017). The traditional crown profile modeling methods have been used to deal with the autocorrelation and heteroscedasticity in the crown profile equations, they are mainly direct variance-covariance modelling (Hann, 1999; Crecente-Campo et al., 2009; Crecente-Campo et al., 2013), mixed-effects modelling (Fu et al., 2013; Sharma et al., 2016; Fu et al., 2017; Sharma et al., 2017; Sun et al., 2017; Jia and Chen, 2019; Wang et al., 2019; Chen et al., 2021; Di Salvatore et al., 2021), and nonlinear marginal modeling (McCulloch and Searle, 2001; Lejeune et al., 2009; de-Miguel et al., 2012; Chen et al., 2022). With the rapid development of machine learning artificial intelligence, some machine learning algorithms have the characteristics of high accuracy and good robustness for the data with nonlinear features (Singh et al., 2016; Dong et al., 2021), which has subsequently been applied to crown profile modeling. Tian et al. (2021) established crown profile model for Chinese fir (*Cunninghamia lanceolata (Lamb.) Hook*) based on random forest algorithm, the accuracy of the random forest model was higher than that of the mathematical model. Chen et al. (2022) recently proposed six machine learning algorithms (MLP, SVR, RF, AdaBoost, GBDT and XGBoost) for the crown profile model of China fir, and found that the performance of the ensemble learning algorithms were superior to single machine learning algorithms and parametric regression approach. However, it appeared that none of the machine learning crown profile modeling methods offered plausible explanation of hierarchical structural features. Here we are facing with space-evolving multidimensional structures (the crown profile database). Deep learning, which refers to machine learning algorithms that construct hierarchical architectures of increasing sophistication (Reichstein et al., 2019), has achieved notable success in modelling ordered sequences and data with spatial context in many fields. Applications to problems in crown profile modeling are in their infancy, but across the key problems (regression, space- or time-dependent data prediction) there are promising. LSTM network can fully explore the internal correlation between time series data, which is specially used to solve the problem of long-term information dependence and avoid gradient disappearance or explosion (Wu, 2019). At present, the application of LSTM model is only based on single factor prediction, and most of them are applied in small sample range. The similarities between the types of data addressed with classical deep learning applications and crown profile data make a compelling argument for the integration of deep learning into the crown profile modelling. There are few reports on the application of crown profile research based on deep learning.

The objectives of this study were to explore the application of deep learning method LSTM and its variant algorithms in the crown profile modeling, using the crown profile database from *Pinus yunnanensis* secondary forests in Yunnan province, in southwest China. It is expected to overcome many of the limitations that have hindered a more wide-spread adoption of machine learning in crown profile modeling problem.

The principal contributions of this paper are as follows: (1) A deep learning prediction based on LSTMs is introduced to explore and exploit the implicit information of hierarchical structural features for crown profile forecasting; (2) To improve the generalization capability and robustness of a single deep learning approach, LSTM’s variant algorithms consisting of a cluster of LSTMs with diverse hidden layers and neurons and LightGBM are developed; (3) A unified framework SHAP were adopted to interprete predictions of ensemble and deep learning models;(4) The performance of the proposed LSTM’s variant algorithms is successfully validated on studies data collected from *Pinus yunnanensis* secondary forests in Yunnan province. Statistical tests of experimental results have demonstrated the proposed LSTM’s variant algorithms is competitive with traditional Vanila LSTM, but substantially outperform ensemble learning model LightGBM.

**Notation**

The following notations (**Table 1**) will be used throughout the remainder of this paper.

**Table 1:** Notation.

Variable	Paraphrase
TH	total tree height in m;
CH	crown height from treetop, 0< H≤TH in m;
DBH	diameter at breast height (1.3 m) in cm;
LCL	largest crown length in m;
RCH	= CH/LCL, relative crown height;
CR	crown radius in m at CH;
CW	crown width in m;
RCR	= CR/LCR, relative crown radius;
TSC	= TH/DBH, tree slenderness coefficients;
CLR	= LCL/TH, crown length ratio;
CFR	=CW/LCL, crown fullness ratio;
HCB	height above ground to crown base in m;
HCW	height above ground to CW in m;

## 2 Materials and methods

### 2.1 Data source and processing

The localities where forest inventories were carried out at Cangshan Mountain(25°34′~26°00′N,99°55′~100°12′E), Yunnan province, SW China, including Malong Peak, Foding Peak and Maer Peak. The three peaks are located on the eastern slope of Cangshan Mountain. The eastern slope of Cangshan Mountain belongs to subtropical climate, with an annual average temperature of 15°C and a dominant wind direction of southwest monsoon. The annual precipitation is abundant, with a rainfall of more than 1000 mm. However, the dry and wet seasons are distinct, and the rainfall is concentrated from May to October, accounting for 84% of the total annual rainfall (Yuan et al., 2008). The predominant tree species are *Pinus yunnanensis*, *Pinus armandii Franch*., and *Tsuga dumosa (D. Don) Eichler.* The typical soil of the area is red soil.

**Figure 1:**
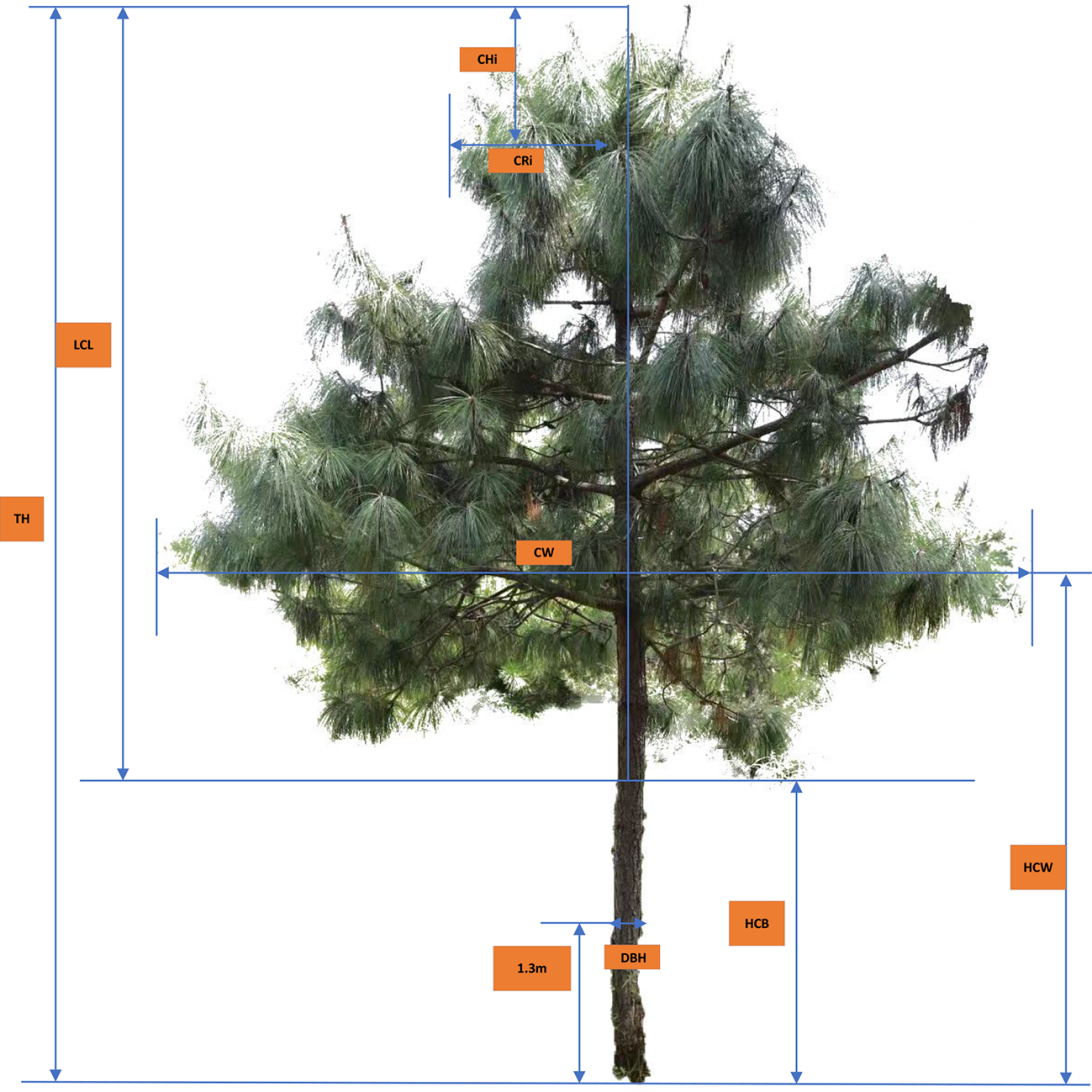
Tree crown measurement diagram of for *Pinus yunnanensis*.

CHi, crown height from treetop to each measurement point i (m); CRi, crown radius to each measurement point i (m).

The predominant tree species *Pinus yunnanensis* were surveyed within three circular sample plots (one plot in each peak). The radius of the corresponding circular sample plot is 18 meters (Malong Peak), 30 meters (Foding Peak) and 20 meters (Maer Peak). In each tree, the crown radius (CR_i_, m) was measured at a different height above crown top (CH_i_, m, i.e., the vertical height from crown top to each crown radius) along the crown profile; the diameter (DBH, cm) was measured at breast height (1.3 m aboveground), to the nearest 0.1 cm, and total tree height (TH, m) was measured to the nearest 0.1 m. The height to the base of the live crown (HCB, m), and the crown width (CW, m, the average values of two measures taken at eastwest direction and north–south direction) were also measured to the nearest 0.1 m in each tree (see **Figure 1**).

The three data files were merged for extensive visual examination, screening, and outlier detection in an initial exploratory analysis before the pooled data were used for modeling. During the exploratory analysis, some obvious data errors were corrected. Tree crown measurements with relative crown height (RCH) than 1 were removed from the data set. Some trees were removed to ensure that each tree crown contained at least four crown radius measurements. Finally, the crown profile data used in this study were collected from 3,096 measured CR values of 516 trees from *Pinus yunnanensis* forests ranging in age from 16 to 45 years. A data summary is presented in **Table 2**.

**Table 2:** Summary statistics of tree characteristic data for 516 sample trees.

Variable	Min	Max	Mean	Std Dev
DBH (cm)	1.8	55.0	15.7	6.4
TH (m)	3.0	19.8	10.4	3.3
CH (m)	0	12.4	2.6	1.9
CR (m)	0	4.7	1.3	0.9
CW (m)	1.4	12.0	5.4	1.8
LCL (m)	0.3	12.4	4.7	2.3
HCB (m)	1.2	10.7	5.7	1.9
HCW (m)	2.0	16.2	6.7	2.0
TSC	0.26	5.72	0.72	0.32
CLR	0.03	0.89	0.44	0.13
CFR	0.38	9.50	1.41	0.94

Considering the multiple measurements for each sample tree crown, the relative crown heights from 0.1 to 1 m with an even interval of 0.1 m were selected to develop crown profile models. However, actual measurements of CH at each tree had non-equidistant space-steps. To overcome this difficulty, the crown profile data were used to obtain numerically interpolated values of CR using piecewise cubic Hermite interpolating polynomial (PCHIP) implemented in MATLAB (R2021b). This interpolation method was adopted because it had the characteristic of preserving shape well. Within each tree crown, *CR_h1_* … *CR_h11_* denote CR in m at the 11 specified relative crown heights (h1 … h30) from crown top, ranging from 0.1 to 1 at an even interval of 0.1.

### 2.2 Multiple factors crown profile modeling based on LSTMs

**Figure 2:**
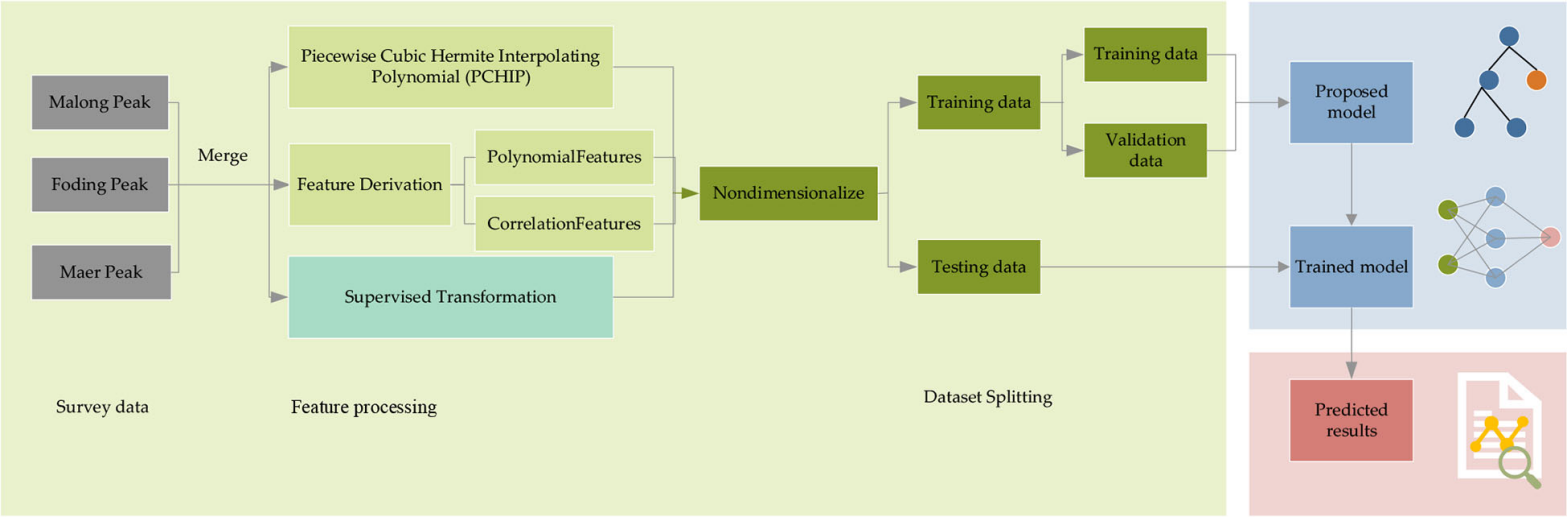
The framework of multiple factors crown profile modeling based on deep learning.

Our research framework is illustrated in **Figure 2**. The whole framework comprises three parts: input features, model, and output. First, we need to extract the features we need from the data we have obtained. Then, we will divide the data into training and testing data, where the training data will be further split the validation data out. Next, the training and validation data are fed into the proposed LSTM and its variant algorithms for training. After the training process, we can put the testing data into the trained model, and it will output the predicted results.

#### 2.2.1 Feature processing

##### 2.2.1.1 Feature derivation

In order to extract the feature information of crown profile data to the maximum extent for LSTM algorithms to train the model, the feature polynomial fusion method and the feature correlation factor method were used to derive new features.

The feature polynomial fusion method can not only get the cross-term feature, but also get the higher order feature. The derivation of polynomial features combines low-dimensional features to obtain high-dimensional features, so that the LSTM models can capture the basic relationship of data to a greater extent and “learn” more data information. We selected the original data features CW, LCL and CH. After the third order polynomial addition, the number of features obtained was 19, of which the number of new features was 16. Feature derived expression were shown in equations (1) and (2).


(1) $$Poly{\left(a,b,c\right)}^{n}=\left(a,b,c,a^{2}ab,ac,b^{2},bc,c^{2},\ldots ,bc^{n-1},c^{n}\right)$$

(2) $$Num_{new}=\sum_{k=1}^{n}\frac{k^{2}+3k+2}{2}-3\;\;\;\;$$

Where *Poly* ()*^n^* is n-order feature derivation; *a,b,c is three* features of dataset; *Num_new_* is the number of *new* derivation features.

For the feature correlation factor method, we defined the following composite tree factors: relative crown height (RCH= CH/LCL), tree slenderness coefficients (TSC= TH/DBH), crown length ratio (CLR= LCL/TH), crown fullness ratio (CFR=CW/LCL). These tree factors were also used to establish crown profile model.

##### 2.2.1.2 Supervised transformation

Since crown profile is influenced by multiple environmental factors, the crown profile can be predicted by the change trend of the continuous adjacent space in each tree. Therefore, in order to fully consider the spatial sequence characteristics affecting the crown profile, CR data at a certain interval was taken as the new feature information, so that the model can fully learn the spatial factors and tree factors of the data. At the same time, the spatial sequence data of samples were combined into pairwise input and output formats, and the spatial sequence prediction problem was transformed into a supervised learning problem.

In this paper, *CR_i-1_* (CR with lag space of 1) was taken as the spatial feature of position *i*, which together with the measured crown factors and the features derived from the features constituted the features of the model.

Finally, the modeled data has 28 input feature variables and one output variable, where the 28 input feature variables include 7 direct measurement features (*DBH, TH, CW, HCW, HCB, LCL, CH*), 16 polynomial features (CW*LCL, CW*CH, LCL^2^, LCL*CH, CH^2^, CW^3^, CW^2^*LCL, CW^2^*CH,LCL^3^, LCL^2^*CW,LCL^2^*CH, CH^3^, CH^2^*CW, CH^2^*LCL, CW*LCL*CH), 4 correlation factor features (*RCH, TSC,CLR, CFR*), and 1 supervised features of transformation (*CR_i-1_*).

##### 2.2.1.3 Non-dimensionalize

Since the data involves multiple indicators that may affect the crown shape, the value range of each indicator is different. In order to unify the impact of index values on the model, this paper used the method of data normalization to make the sample data non-dimensionalize, and the normalized mathematical expression was equation (3).


(3) $$x^{\prime }=\frac{x-min}{max-min}$$

Where, ***x*** is the data value of an index before dimensionalization, max and min are respectively the maximum and minimum values of this index in all samples. ***x****’* is the non-dimensionalized data value of this index.

#### 2.2.2 LSTM algorithms and modeling scheme

**Figure 3:**
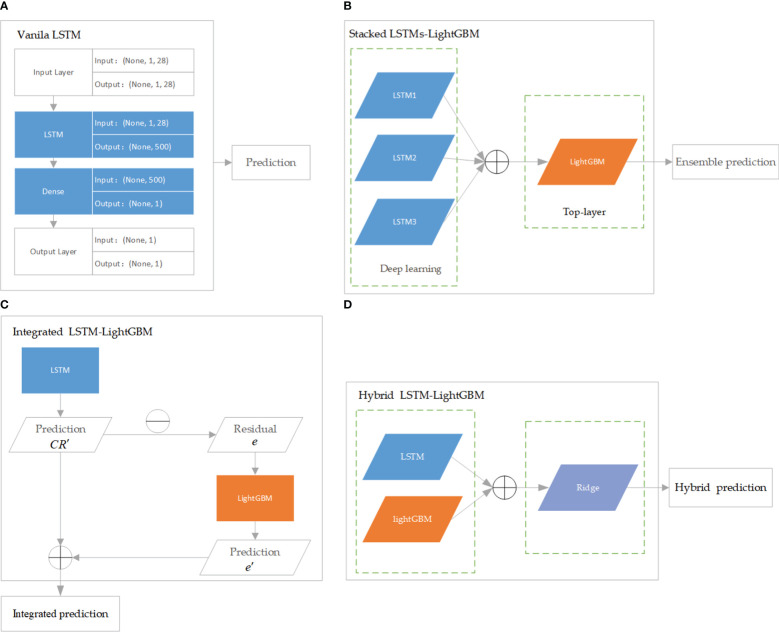
The architecture of LSTM and its variant algorithms. **(A)** is Vanila LSTM algorithm; **(B)** is Stacked LSTMs-LightGBM algorithm; **(C)** is Integrated LSTM-LightGBM algorithm; **(D)** is Hybrid LSTM-LightGBM algorithm.

LSTM is a modification of recurrent neural networks (RNNs) – neural networks that allow feedback loops to communicate data from a node in a forward layer to a node in a backward layer (Géron, 2022). LSTM networks overcome the problem of vanishing and exploding gradient problems by intelligently forgetting some past irrelevant information, and hence such network proves very suitable for modeling sequential data (Mehtab et al., 2020). LightGBM is a recent modification of the GB algorithm. It can outperform existing boosting frameworks on both efficiency and accuracy, with significantly lower memory consumption (Ke et al., 2017). JoZefowicz et al. (2015) analyzed the performance of more than 10,000 different LSTM permutations, some from the literatures (Krause et al., 2016; Chen et al., 2018; Kent and Salem, 2019; Lu et al., 2020) but most generated as LSTM variants, and found that some of the mutations did perform better than the classic LSTM, but not all, of the tasks, studied. In this study, the regression models based on the crown profile data sets were developed using LSTM and its variant algorithms, namely, stacked LSTMs-LightGBM, integrated LSTM-LightGBM, and hybrid LSTM-LightGBM. The models were optimized and extensively evaluated with the following modeling scheme. Vanila LSTM: Vanilla LSTM is defined as three parts: input layer, LSTM hidden layer, and fully connected output layer. We implemented a one-hidden-layer LSTM model using Keras with the Tensorflow backend (**Figure 3A**). The model hyperparameters were optimized within the predefined range: number of hidden units (100−1000), epoch (50−200), learning rate (0.0001,0.001,0.01), size of minibatch (10-100), optimization algorithm (*Adam ()*), activation function (*relu* for hidden layers), dropout rates for hidden layers (0, 0.1, 0.2).

Stacked LSTMs-LightGBM: The proposed ensemble deep learning method consisted of three LSTMs and LightGBM was developed in this paper. More specifically, the LightGBM aggregated the outputs of different LSTMs. In the structure of stacked LSTMs-LightGBM, the predictions of a cluster of LSTMs were input into a LightGBM regression top-layer to produce the final forecasting (**Figure 3B**). The performance of ensemble learning depended vastly on the parameters of top-layer. Three hyperparameters governing the performance of the LightGBM regressor were optimized within the following number of leaves (“*num_leaves*”, 50−500), number of iterations (“*n_estimators*”,30−900), and learning rate (“*learning_rate*”, 10^−4^−10^−1^).

Integrated LSTM-LightGBM: Integrated LSTM-LightGBM was a combination of LSTM and LightGBM models to give full play to the advantages of combined model prediction. After the LSTM model was used to predict CR, LightGBM model was used to introduce the relaxation variable method to correct each predicted CR value for the calculated residual *e* (**Figure 3C**). The predicted value of LSTM model (*CR^’^*) and the error corrected by LightGBM (*e’*) were integrated to obtain the prediction result of Integrated LSTM-LightGBM, namely ( $\hat{CR}$ = *CR^’^* + *e’*). LSTM parameters adopted the optimized parameters of the Vanila LSTM model, and LightGBM hyperparameter range was defined as follows: number of leaves (“num_leaves”, 10−200), number of iterations (“n_estimators”,30−300), and learning rate (“learning_rate”, 10^−3^−10^−1^).

Hybrid LSTM-LightGBM: The Hybrid LSTM-LightGBM took the predicted CR value of LSTM and LightGBM models as a feature input, and the measured CR value as an output. Ridge regression was performed, and the final hybrid model prediction result was obtained according to the obtained Ridge regression model (**Figure 3D**). The *linear_model.RidgeCV* function from scikit-learn was used to develop the regression models with hyperparameter optimized within the predefined ranges suggested: alphas (10^−6^−10).

The specific definitions of the relevant hyper-parameters are described in the Supplementary_Material.docx

### 2.3 Performance evaluation

The performance of the proposed algorithms was evaluated based on their balanced the determination coefficient (R^2^, equation (4)), the mean squared error (MSE, equation (5)), the mean absolute deviation (MAE, equation (6)), and the Mean Deviation (ME, equation (7)) values. The computation time of the proposed algorithms was also recorded to indicate the speed of each method.


(4) $$R^{2}=1-\frac{\sum_{i=1}^{n}{\left(y_{i}-{\overset{\wedge }{y}}_{i}\right)}^{2}}{\sum_{i=1}^{n}{\left(y_{i}-\bar{y_{i}}\right)}^{2}}$$

(5) $$MSE=\frac{1}{n}\sum_{i=1}^{n}{\left(y_{i}-\overset{\wedge }{y_{i}}\right)}^{2}$$

(6) $$MAE=\frac{1}{n}\sum_{i=1}^{n}\left|y_{i}-\overset{\wedge }{y_{i}}\right|$$

(7) $$ME=\frac{\sum_{i=0}^{n}\left(y_{i}-\hat{y_{i}}\right)}{n}\;$$

where *y_i_* represents the observed value for the ith analytic tree *i*^th^; $\overset{\wedge }{y_{i}}$ is the predicted value of *i^th^* observed value; *n* is the number of trees, $\bar{y_{i}}$ is the mean value for the observed.

The data split ratio adopted by the test was 8:1:1. That is, 80% of original data were used as the training set to train the model parameters, 10% of the original data was used as the validation set, which was used for model optimization during model training, and another 10% of original data was used as the test set to test the forecasting effect of model (Ju et al., 2019). All of models were run on a computer with Windows 11 operating system, Intel Core i7 CPU @ 3.20 GHz and RAM of 16.00 GB.

Understanding why a model makes a certain prediction can be as crucial as the prediction’s accuracy in the research of machine learning modeling. Here, we used SHAP (Lundberg and Lee 2017) to interpret the predictions of ensemble or deep learning models. We calculated SHAP value using *shap* package in Python. SHAP overall process: (1) Select the training set as the basic data set, and the explanation model instantiates an interpreter; (2) Select the test set as the explanation sample, and then calculate the SHAP value corresponding to each feature of each sample in the explanation sample through the interpreter. The greater the SHAP value, the greater the feature contribution.

## 3 Results

### 3.1 Prediction of the crown profile model

The parameters used in all the models were tuned by grid search or experiment, and the parameters of the ensemble learning model were the same as the parameters of the single model, which ensured the validity of ensemble learning. The optimizing parameters can ensure that all of parameters maintain the good performance in training and prediction. The optimized parameters of all the models used in the experiment are summarized in **Table 3**.

**Table 3:** The parameter values for different models.

Model	Python Package	Hyper-parameter Value
Vanila LSTM	from keras.models import Sequential from keras.layers import LSTM from keras.layers import Dense	units =500, epochs=100, batch_size=10, optimizer=‘adam’, learning rate=0.0001 recurrent_activation = ‘sigmoid’
Stacked LSTMs-LightGBM	from keras.models import Sequential from keras.layers import LSTM from keras.layers import Dense import lightgbm as lgb	(the same LSTM parameter values as mentioned above) num_leaves=5, learning_rate=0.1, n_estimators=49
Integrated LSTM-LightGBM	from keras.models import Sequential from keras.layers import LSTM from keras.layers import Dense import lightgbm as lgb	(the same LSTM parameter values as mentioned above) num_leaves=42, learning_rate=0.05, n_estimators=23
Hybrid LSTM-LightGBM	from keras.models import Sequential from keras.layers import LSTM from keras.layers import Dense import lightgbm as lgb from sklearn import linear_model	(the same LSTM parameter values as mentioned above) num_leaves=29, learning_rate=0.1, n_estimators=56 alphas=0.1

**Figure 4:**
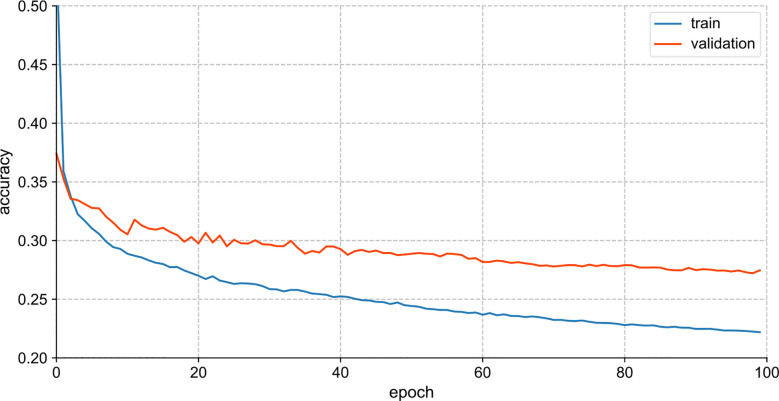
Accuracy and loss curves of training set and verification set (Vanila LSTM model).

**Figure 4** visualizes the training performance of Vanila LSTM. For Vanila LSTM model, the loss curves of the training and validation sets tended to be flat after parameter tuning, and the learning rate decreased to 0.0001. In the case of not amplifying the samples of the dataset, the model was fully trained at this time, but the effect of the training model had room for further improvement.

**Figure 5:**
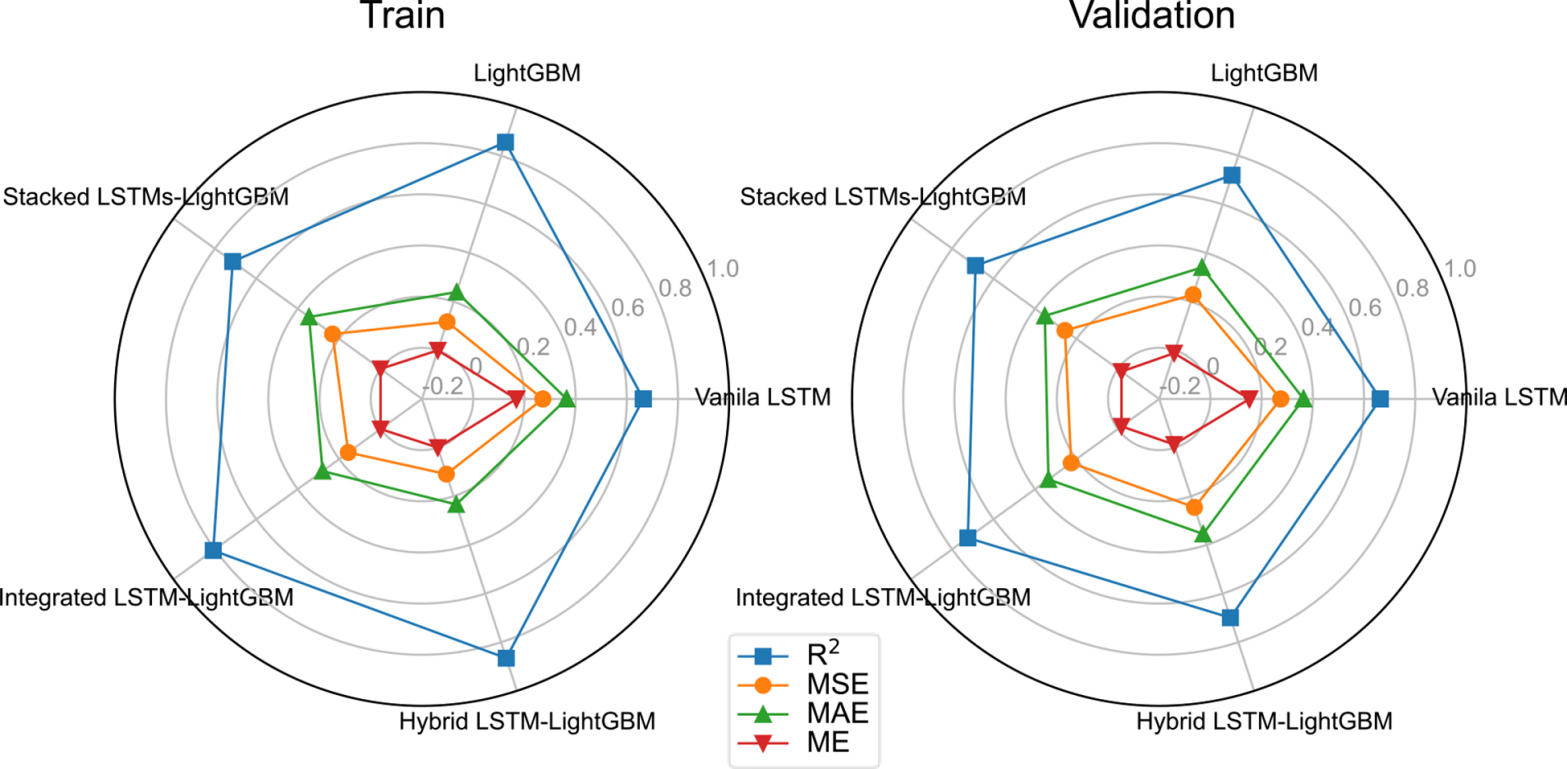
Performance Evaluation for the five algorithms on the validation and test subsets.

The variant algorithm of LSTM has been used to further improve model accuracy. The training results of different crown profile models based on LSTM and its variant algorithms are listed **Table 4**. We observed that the LSTM’s variant algorithms performed higher accuracy compared to molecular LSTM algorithm (Vanila LSTM). From **Table 4** and **Figure 5**, the proposed Hybrid LSTM-LightGBM performs better than two other variant algorithms with the minimum value of MAE as 0.2336, MSE as 0.1089 and ME as 3.91E-16 and the maximum value of R^2^ as 0.8656 for training datasets. And the better one of two other variant algorithms is Integrated LSTM-LightGBM with MAE as 0.2798, MSE as 0.1558, ME as -5.49E-12 and R^2^ as 0. 8078.For validation datasets, the proposed Integrated LSTM-LightGBM realizes the best rank, followed by Hybrid LSTM-LightGBM. Moreover, from the analysis of training time, the Hybrid LSTM-LightGBM required slightly more computational time compared to Integrated LSTM-LightGBM. We also observed that LightGBM was the fastest algorithm among the proposed algorithms as it consumed the shortest computation time for training datasets in this study, whereas Stacked LSTMs-LightGBM was the most time-consuming algorithm, requiring, on average, approximately 719 times longer computational time than LightGBM due to large number of algorithm parameters (**Table 4**).

**Table 4:** The training results of different crown profile models based on LSTM and its variant algorithms.

Model	Train					Validation			
	R^2^	MSE	MAE	ME	Training time	R^2^	MSE	MAE	ME
Vanila LSTM	0.6643	0.2721	0.3650	0.1689	126.16	0.6637	0.2746	0.3634	0.1524
LightGBM	0.8554	0.1172	0.2396	4.75E-10	**0.48**	0.7199	0.2287	0.3407	**-0.0117**
Stacked LSTMs-LightGBM	0.7139	0.2319	0.3454	3.69E-10	344.94	0.6871	0.2555	0.3524	-0.0178
Integrated LSTM-LightGBM	0.8078	0.1558	0.2798	-5.49E-12	126.36	**0.7242**	**0.2252**	**0.3351**	-0.0178
Hybrid LSTM-LightGBM	**0.8656**	**0.1089**	**0.2336**	**3.91E-16**	126.64	0.6994	0.2455	0.3549	-0.0128

Best performance is highlighted in bold.

**Table 5** shows the error for different model predictions. As seen from the four forecasting performance indices (R^2^, ME, MAE and MSE) that the Integrated LSTM-LightGBM model had some degrees of advantages over LightGBM and Vanila LSTM, indicating that in the case of more complex data, LSTM’s variant algorithms were sufficient to learn crown profile features and it could predict accurately. Although the ensemble learning model (LightGBM) was not inferior to Vanila LSTM and its other variant algorithms in test results, the overall trend of the deep learning model was better. The comparable model performance on both validation and test sets indicated that the applied LSTM’s variant algorithms offered generalizability and transferability of the developed model to previously LSTM algorithm. Rank metric from testing results showed that Integrated LSTM-LightGBM performance achieved the best performance followed by LightGBM and Stacked LSTMs-LightGBM.

**Table 5:** Comparison of test results of different crown profile models based on LSTM and its variant algorithms.

Model	R^2^ (Rank)	MSE (Rank)	MAE (Rank)	ME (Rank)	Sum Rank (Rank)
Vanila LSTM	0.6692 (5)	0.2942 (5)	0.3959 (5)	0.2028 (5)	20 (5)
LightGBM	0.7197 (2)	0.2493 (2)	0.3586 (2)	0.0607 (3)	9 (2)
Stacked LSTMs-LightGBM	0.7040 (3)	0.2633 (3)	0.3700 (3)	**0.0379 (1)**	10 (3)
Integrated LSTM-LightGBM	**0.7308 (1)**	**0.2394 (1)**	**0.3537 (1)**	0.0458 (2)	**5 (1)**
Hybrid LSTM-LightGBM	0.6900 (4)	0.2757 (4)	0.3780 (4)	0.0664 (4)	16 (4)

Rank is the rating of the test statistic (R^2^, ME, MAE and MSE), the smaller the rating value, the better the model prediction result. the best performance is highlighted in bold.

### 3.2 Relative importance of influential predictors

**Figure 6:**
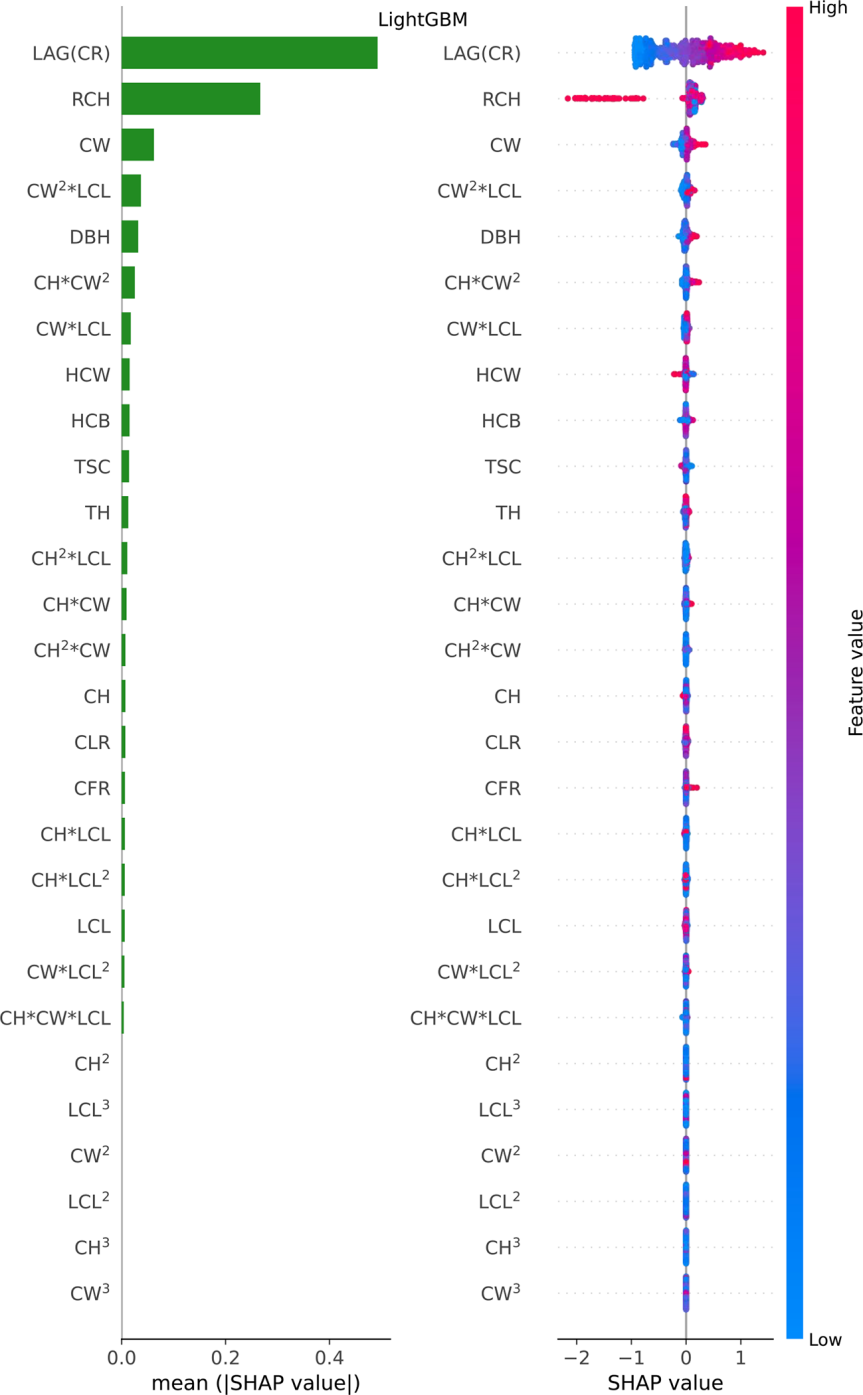
Feature importance bar plot (mean (|SHAP value|), left plot) and beeswarm plot (mean value, right plot) for LightGBM model. Where color represents characteristic value (red high, blue low). * indicates a multiplication sign.

**Figure 7:**
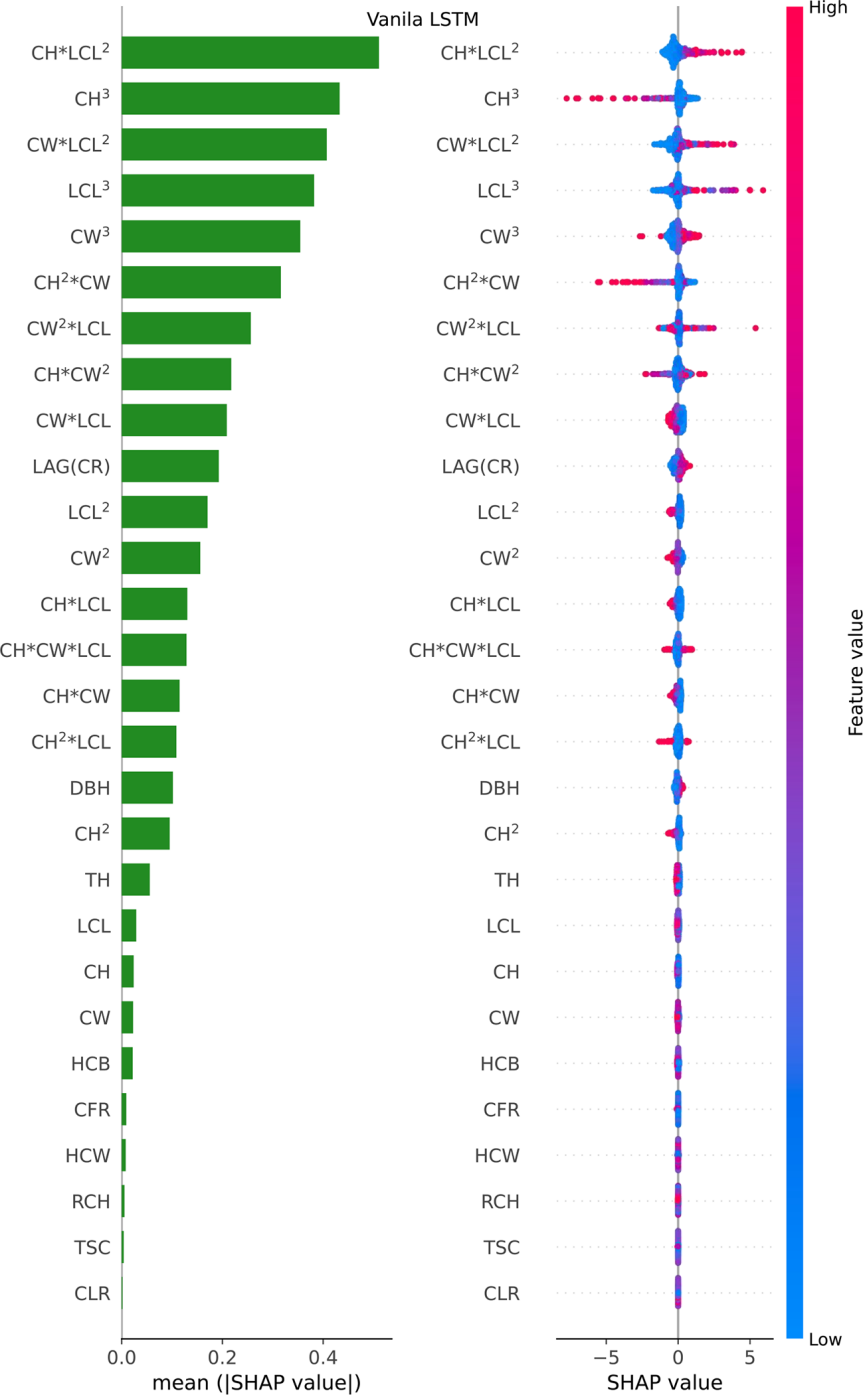
Feature importance bar plot (mean (|SHAP value|), left plot) and beeswarm plot (mean value, right plot) for Vanila LSTM model. Where color represents characteristic value (red high, blue low). * indicates a multiplication sign.

Considering that LSTM’s variant algorithms were composed of LightGBM and Vanila LSTM, the relative importance of potential influential predictors for predicting crown profile was investigated for each of LightGBM (**Figure 6**) and Vanila LSTM models (**Figure 7**). For beeswarm plot, each point is a result, its position on the x-axis represents the SHAP value of the feature, and the color represents the relative size of the feature, with red representing high and blue representing low.

For LightGBM model, LAG(CR) (*CR_i-1_*), RCH and CW were found the most important predictors, while other variables were relatively insignificant variables (see **Figure 6** feature importance bar plot). Furthermore, we found that LAG (CR), the most important feature, was basically positively correlated with CR (the model output); The RCH also had an obvious influence. Further analysis, if the RCH is large, the estimated value will be reduced significantly, because the leftmost point in the row of RCH is basically red in feature importance beeswarm plot; The SHAP value in the data point with large CW value is greater than 0, which belongs to positive correlation (see **Figure 6** beeswarm plot).

For the Vanila LSTM model, the most important variables were found to be the Poly (*CW, LCL, CH*)^3^ and the original measurement tree factors (LAG(CR), DBH and TH), and the least important predictors were some derived factor (CLR, TSC, RCH, HCW, CFR and HCB), in that order (see **Figure 7** left plot). The beeswarm plot (see **Figure 7** right plot) shows that CH*LCL^2^, CW*LCL^2^, LCL^3^ and CW^2^*LCL are obviously positively correlated with predictors, while CH^3^ and CH^2^*CW are basically negatively correlated with predictors. The blue dots of CH*LCL^2^, CW*LCL2, LCL^3^ and CW^3^ variables are mainly concentrated in the area where SHAP value is less than 0, indicating that when their values are small, the estimate of CR will be reduced. For feature DBH, CH, LCL and CW, most of the points are diffuse in SHAP=0, so it has no effect on most of them, only a small part.

The relative importance of influential predictors was different for the LightGBM and Vanila LSTM models. There were only three factors that contribute significantly to LightGBM model, while almost all factors contributed to Vanila LSTM model. This phenomenon can explain why deep learning outperforms ensemble learning when there are many features.

## 4 Discussion

### 4.1 Performance and comparison of models

Previous studies have shown that machine learning has broad application in crown profile modeling. These machine learning includes MLP, SVR, RF, AdaBoost, GBDT and XGBoost (Tian et al., 2021; Chen et al., 2022). Among them, the ensemble learning algorithms can deal with complex nonlinear relationship and show strong prediction ability when predicting the crown profile (Chen et al., 2022).The purpose of this paper is to find the applicable model for crown profile prediction by comparing the ensemble and deep learning algorithms based on the same data format. So far, the deep learning algorithm has not been used to predict crown profile yet based on space-dependent data. Results from this study (see **Table 4** and **Table 5**) showed that the Integrated LSTM-LightGBM model consistently obtained the best performance for crown profile prediction compared with LightGBM model. The overall trend of the deep learning model was better than the ensemble learning model.

An important finding was that there were only three feature variables that contribute significantly to LightGBM model, while almost all feature variables contributed to Vanila LSTM model (see **Figures 6**, **7**). Two phenomena can be attributed to the finding. Firstly, the LSTM’s variant model was superior to the single LSTM model. The model relied on feature variables to develop its prediction algorithms. So, when the number of features contributed to model was less, the generalization ability of the whole model was therefore reduced and the accuracy of the predictor importance measurement was also affected. Secondly, the deep learning outperformed ensemble learning when there were many features (Zhang et al., 2018; Jan et al., 2019). The LSTM series was affected by more variables than LightGBM in this study. This finding supported the notion that more feature variables bring more room for improvement for LSTM series.

### 4.2 The interpretability of ensemble or deep learning models

Previous results have shown that the machine learning is an effective technique in improving the crown profile prediction accuracy. However, understanding why a model makes a certain prediction can be as crucial as the prediction’s accuracy (Lundberg and Lee, 2017). Interpretable machine learning techniques can generally be grouped into two categories: intrinsic interpretability and post-hoc interpretability (Du et al., 2019). Intrinsic interpretability incorporates interpretability directly to their structures, including decision tree, rule-based model, linear model, attention model, etc. In contrast, the post-hoc interpretability requires selecting and training a black-box model (ensemble or deep learning) and applying interpretability methods (feature importance, partial dependency graph) to explanate after training (Molnar, 2020). Current explanations are usually given in the format of feature importance vectors, which are a complete causal attribution explanation (Molnar et al., 2018). The explanation audiences, such as developers or researchers, can utilize the statistical analysis of the feature importance distribution to debug the models (Du et al., 2019).

In this paper, we adopt a unified framework for interpreting predictions of ensemble or deep learning models, SHAP. The relative importance of influential predictors was different for the LightGBM and Vanila LSTM models. There are more factors that contribute significantly to Vanila LSTM model compared to LightGBM model. The phenomenon can be attributed to two causes. Firstly, over-fitting can be a problem, especially for the LightGBM model. The generalization ability of the whole model was therefore reduced and the accuracy of the predictor importance measurement was also affected. Secondly, the problem of non-convex optimization makes convergence to a local optimum possible when the parameters of the model are learned and adjusted. This will lead to the deviation of the predictor importance’s estimation (Huang et al., 2019).

### 4.3 Limitations and further research

One of the limitations of this paper is that whether it is LightGBM or LSTM series, whether it is a Vanila LSTM model or an LSTM’s variant model, their robustness is difficult to guarantee for abnormal data and false data, but compared to the ensemble and deep learning models, the model generated by deep learning is still better, but it will consume more computing resources. Fortunately, the computational complexity of these algorithms is relatively small in crown profile modelling, and the main purpose of this paper is to explore the performance of deep learning methods. However, if we can solve the above problems, it will be more conducive to the improvement of the algorithm, this part of the task will be placed in our follow-up work.

Age has a certain influence on the crown profile (Yu et al., 2021). However, age variable inputs have not been used in this paper to predict crown profile because it is difficult to obtain the age of each tree for the uneven aged forest in this study. In future research, age variable may be used for improving the model predictions of crown profile. Moreover, current models may be biased when applied at a large scale as the crown profile is largely influenced by site quality, stand density, spatial structure (such as mingling, neighborhood comparison, and uniform angle index), and random variabilities caused by various stochastic factors that vary from stands to stands. Thus, the prediction bias can be reduced through the integration of all kinds of variability into the crown profile models (Sharma et al., 2017).

Several important model-agnostic interpretability methods such as Partial Dependence Plot (PDP), Individual Conditional Expectation (ICE), Permuted Feature Importance, Global Surrogate, Local Surrogate (LIME), SHAP Value exist, and while none of them are perfect, they can help researchers interpret the results of even very complex machine learning models (Zhou, 2019). For this study, we only used a method (SHAP Value) to interpret the predictions of ensemble or deep learning models. In the future work, multiple model-agnostic methods may be adopted, each of them represents a step toward more fully understanding machine learning models. As machine learning becomes more and more ubiquitous, grasping how these models find answers will be crucial to improving their performance and reliability.

## 5 Conclusions

This paper has introduced a novel method using deep learning prediction based on LSTMs, LightGBM and Ridge algorithm for crown profile forecasting. In the proposed LSTM’s variant models, a cluster of LSTMs with diverse hidden layers and neurons are employed separately to learn the information of crown profile. When compared with proposed prediction models including Vanila LSTM, LightGBM, Stacked LSTMs-LightGBM, Integrated LSTM-LightGBM and Hybrid LSTM-LightGBM, the pro-posed Hybrid LSTM-LightGBM can achieve a best forecasting performance with the minimum value of MAE, MSE and ME and the maximum value of R^2^ for training datasets, and the proposed Integrated LSTM-LightGBM can achieve a best forecasting performance on both validation and test sets. Furthermore, the analysis of feature importance of LightGBM and Vanila LSTM present that there are more factors that contribute significantly to Vanila LSTM model compared to LightGBM model. This phenomenon can explain why deep learning outperforms ensemble learning when there are more interrelated features. In conclusion, the following are the highlights of the study:

(1) LSTM’s variant algorithms obtained the best performance for crown profile prediction.

(2) The overall trend of the deep learning model was better than the ensemble learning model.

(3) We used a method (SHAP Value) to interpret the predictions of black-box models (ensemble or deep learning).

## Data availability statement

The original contributions presented in the study are included in the article/**Supplementary Material**. Further inquiries can be directed to the corresponding author.

## Author contributions

YC and JW contributed to the study design and performed the formal analysis. YC performed the software analysis and wrote the first draft of the manuscript. JW contributed data curation. YC and JW contributed to the writing, review, and editing. All authors contributed to the article and approved the submitted version.
