# Supplementary material for: Deep learning for crown profile modelling of Pinus yunnanensis secondary forests in Southwest China
Source: Front Plant Sci. 2023 Feb 3;14:1093905. doi: 10.3389/fpls.2023.1093905 (PMC9936141; doi:10.3389/fpls.2023.1093905)
Supplement: Supplementary file 1 [file Table_1.docx]

Supplementary Material

# Hyper-parameter

Hyper-parameter tuning can largely affect the predictive performance of machine learning algorithms (Feurer et al.,2015). Setting a suitable configuration for the hyper-parameters of a machine learning algorithm is usually performed by trial and error（Mantovani et al.,2017）. Depending on the training time of the machine learning algorithm used, finding a good set of values manually can be very time-consuming. As a result, recent works in hyper-parameter for machine learning algorithms focus on the development of better hyper-parameter tuning techniques (Bergstra et al.,2011,2013).

the definition of hyper-parameters

- **units**: Positive integer, dimensionality of the output space.
- **inputs**: A 3D tensor with shape [batch, timesteps, feature].
- **recurrent_activation**: Activation function to use for the recurrent step.
- **n_estimators:** The number of trees in the forest.
- **learning_rate**: Learning rate shrinks the contribution of each tree by learning_rate. There is a trade-off between learning_rate and n_estimators. Values must be in the range (0.0, inf).
- **num_leaves**: max number of leaves in one tree. aliases: num_leaf, max_leaves, max_leaf, max_leaf_nodes, constraints: 1 < num_leaves <= 131072
- **alphas**: ndarray of shape (n_alphas,), Array of alpha values to try. Regularization strength; must be a positive float. Regularization improves the conditioning of the problem and reduces the variance of the estimates. Larger values specify stronger regularization. Alpha corresponds to 1 / (2C) in other linear models such as LogisticRegression or LinearSVC. If using Leave-One-Out cross-validation, alphas must be positive.

References

Feurer, M., Springenberg, J., & Hutter, F. (2015). Initializing bayesian hyperparameter optimization via meta-learning. In Proceedings of the AAAI Conference on Artificial Intelligence 29(1).

Bergstra, J., Bardenet, R., Bengio, Y., & Kégl, B. (2011). Algorithms for hyper-parameter optimization. Advances in neural information processing systems, 24.

Bardenet, R., Brendel, M., Kégl, B., & Sebag, M. (2013). Collaborative hyperparameter tuning. In International conference on machine learning. PMLR. 28(2):199-207

Mantovani, R. G. , Tomá Horváth, Cerri, R. , Vanschoren, J. , & André C P L F De Carvalho. (2017). Hyper-Parameter Tuning of a Decision Tree Induction Algorithm. Intelligent Systems. IEEE.
